# The Practical Medicine Series of Year Books

**Published:** 1902-03

**Authors:** 


### BOOK REVIEWS.

The Practical Medicine Series of Year Books.—Comprising Ten Volumes of the Year's Progress in Medicine and Surgery. Volume III., Eye, Ear, Nose and Throat. Edited by Casey A. Wood, C.M., M.D., Albert H. Andrews, M.D., T. Melville Hardie, A.M., M.D.

This volume is intended to take the place of the Year Book on the Ear, Nose and Throat which had previously been published by Drs. Andrews and Head. In the new volume the Eye is also included. The task of collecting and collating those articles in medicine and surgery which have been of special importance is exceedingly difficult. The authors in this work have succeeded most admirably. Such a work as this is certainly of great value to those who have not a ready access to a large medical library, and who are also unable to read extensively all the yearly medical literature bearing upon the subject in question. The only criticism that we would make of this new volume is the appearance ot cheapness in the general make-up of the paper and binding. It certainly does not impress one as on the same equality as the former Year Books. However, this volume has distinct value. Roy.

